# Assessment and quantification of ipsilateral and contralateral ankle joint alignment changes after unilateral total knee arthroplasty for knee osteoarthritis with varus deformity: a retrospective cohort study

**DOI:** 10.1186/s43019-025-00277-2

**Published:** 2025-05-23

**Authors:** Ahmed A. Khalifa, Amr A. Fadle, Abdelrahman A. Aziz Khalaf, Ahmed M. Abdelaal, Mohamed M. A. Moustafa

**Affiliations:** 1 Orthopedic and Traumatology Department, Qena Faculty of Medicine and University Hospital, South Valley University, Kilo 6 Qena-Safaga Highway, Qena, 83523 Egypt; 2 Orthopaedic and Traumatology Department, Assiut University Hospital, Assiut, Egypt

**Keywords:** Total knee arthroplasty, TKA, Ankle joint, Realignment, Primary osteoarthritis, Varus deformity

## Abstract

**Purpose:**

The study’s primary objective was to assess and quantify the ipsilateral (side A) and contralateral (side B) ankle joint line orientation (AJLO) changes after unilateral total knee arthroplasty (TKA) for primary knee osteoarthritis (OA) with varus deformity. The secondary objectives were to detect if there was a correlation between the knee deformity correction and AJLO changes and if the knee and ankle clinical outcomes on the TKA side correlate with joint alignment changes.

**Methods:**

This retrospective cohort study included 70 patients with a mean age of 61.76 ± 5.96 years. The lower limb alignment was evaluated using the hip-knee-ankle (HKA) angle, while the AJLO was assessed using the tibial plafond to horizontal line angle (TPHA). The functional outcomes for side A were evaluated at a median follow-up of 18 (interquartile range (IQR): 12–46.2) months using the Knee Injury and Osteoarthritis Outcome Score (KOOS) and The American Orthopaedic Foot and Ankle Society (AOFAS) score for the knee and ankle joints, respectively.

**Results:**

In side A, the HKA significantly improved from 167.49 ± 6.25 to 177.08 ± 4.39 (*p* < 0.001). No difference in AJLO was found between both sides preoperatively (*p* = 0.329). At the last follow-up, in side A, the AJLO changed significantly into less varus from −7.11 ± 5.44° to −1.10 ± 4.91° (*p* < 0.001); in side B, the AJLO showed no significant changes (−6.38 ± 6.10° versus −6.65 ± 6.50°, *p* = 0.970). For side A, the KOOS and AOFAS showed significant improvement, 45.20 ± 14.94 versus 75.72 ± 13.28 (*p* < 0.001) and 70 (65–75) versus 90 (80–90; *p* < 0.001), respectively. The preoperative HKA and AJLO on side A and side B showed significant positive correlations (*r* = 0.591, *p* < 0.001 and *r* = 0.611, *p* < 0.001, respectively). On side A, the postoperative HKA and AJLO showed a significant positive correlation (*r* = 0.298, *p* = 0.012). The preoperative and postoperative AJLO and AOFAS on side A showed nonsignificant negative correlations (*r* = −0.202, *p* = 0.277 and *r* = −0.115, *p* = 0.537, respectively). The preoperative and postoperative HKA and AOFAS on side A showed nonsignificant positive correlations (*r* = 0.126, *p* = 0.499 and *r* = 0.331, *p* = 0.069, respectively). The linear regression analysis indicated that for every 1° correction in HKA, the AJLO changed by 0.5° (*R*^2^ = 0.241, 95% confidence interval (CI) 0.298–0.747, *p* < 0.001).

**Conclusions:**

The ipsilateral ankle joint realigned to a less varus position after ipsilateral TKA for managing knee OA with varus deformity, with an estimated half a degree of less varus AJLO after HKA correction by a degree. No changes occurred in the contralateral ankle joint. The ankle joint function improvement was not correlated to the HKA or AJLO changes.

## Introduction

Although total knee arthroplasty (TKA) is one of the most successful surgeries for managing end-stage knee diseases with acceptable long-term outcomes [1–3], inferior clinical outcomes and lower patient satisfaction after TKA are still being reported, where reasons outside the knee joint (extra-articular causes) were suggested, such as the presence of ankle osteoarthritis (OA) and its aggravation after TKA and the inability of the ankle and hindfoot to compensate for the knee deformity correction post-TKA, leading to pain in patients with normal ankle and foot joints [4–11].

To avoid such causes, investigating the relationship between ankle and foot realignment after knee deformity correction (either by TKA or around-the-knee osteotomies) has been an area of research interest for the past few years [9, 10, 12–16]. Correcting the knee varus deformities will lead to a relative shift of the weight-bearing line from medial to lateral with subsequent effect on the ankle joint, leading to changes in the tibiotalar contact surface area and intraarticular ankle pressure changes as proved in cadaveric studies [16, 17]. Furthermore, it might lead to aggravated ankle pain in patients with preexisting ankle OA [10, 18–20].

Assessing and anticipating the ankle joint realignment and adaptation before TKA surgery would help in processing the preoperative planning and patient counseling regarding expecting some changes not only at the knee level but also at the ankle and foot joints [21]. Few authors proposed prediction models to calculate the amount of possible ipsilateral ankle joint realignment after correcting knee varus deformities by TKA [21–23]; however, as the operated side possesses some anatomical changes after deformity correction, such as changes in the lower limb length [24, 25], we assumed that possible adaptation might occur in the contralateral ankle joint on the non-operated side, which was rarely evaluated in literature [26].

Therefore, the primary objective was to assess and quantify the ipsilateral and contralateral ankle joint line orientation (AJLO) changes after unilateral TKA to correct knee varus deformity associated with primary knee osteoarthritis. We aimed to evaluate the following as secondary objectives: (1) whether there was a correlation between the knee deformity correction and ankle joint line realignment and (2) whether the knee and ankle clinical outcomes on the TKA side correlate with joint alignment changes.

## Methods

This retrospective cohort study was performed following the ethics standards according to the Declaration of Helsinki and after being approved by our institution’s ethical committee review board.

We included patients diagnosed with bilateral primary knee OA with varus deformities who underwent unilateral TKA with at least 6 months of follow-up and with ankle joints on both sides appearing normal in the radiographs. We excluded patients who had revision TKA, patients with simultaneous bilateral TKA, those who had an extraarticular deformity (previous fractures or corrective osteotomies), patients with apparent ankle arthritis or deformities (secondary to fractures or Charcot arthropathy), and patients with radiographs that were not obtained per the study protocol (such as those with limb malrotation and if both ankles were not included). The knees of the included patients were divided into side A (the side that underwent TKA) and side B (the non-operated side).

Besides basic physical assessment, all patients had preoperative long (hip-knee-ankle) anteroposterior (AP) plain radiographs as part of the preoperative planning. The radiographs were performed following the same protocol, with both knees facing forward in a bipedal stance and a constant distance between the feet while the patient was standing on a solid radio-opaque board (serving as a mark for the horizontal plane reference). The degree of OA was evaluated in the radiographs and classified according to the Kellgren and Lawrence (KL) grading system [27].

### Surgical details

The perioperative protocol was the same for all patients. All surgeries were performed by fellowship-trained surgeons following the same technique and steps [28, 29]. Under spinal anesthesia, while a tourniquet was applied to the upper thigh, surgeries were carried out through a medial parapatellar approach, adopting the measured resection philosophy aiming eventually for a neutral mechanical alignment after obtaining proper soft tissue balancing. The distal femoral cut was carried out using an intramedullary alignment rod, and the resection angle was adjusted according to the valgus correction angle measured in the preoperative radiographs. The tibial resection was performed using either an intramedullary or extramedullary alignment system (per surgeon preference). The patella was not resurfaced in any of the patients, and in all cases, a cemented posterior stabilized knee prosthesis was used. The postoperative care and rehabilitation were the same for all patients per our institution’s protocol [2, 30].

### Outcomes assessment

The desired outcomes were collected for all patients preoperatively and compared with the last follow-up evaluations; if a patient was scheduled for contralateral-side TKA, the outcomes were collected before the procedure.

**Fig. 1:**
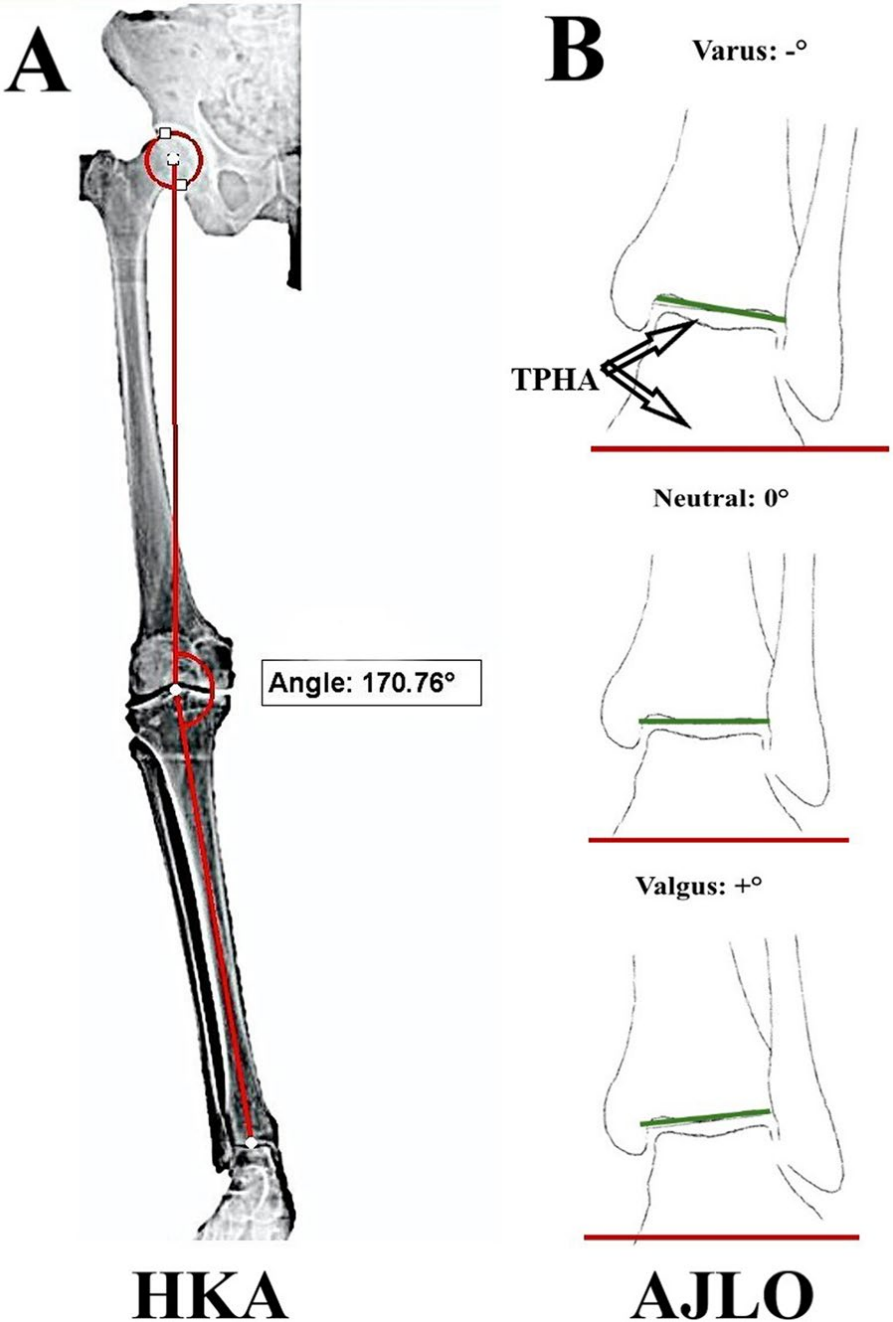
Schematic illustration for the radiological parameter measurements. **A** The hip-knee-ankle (HKA) angle is the medial angle between the femoral and tibial mechanical axes (red lines). **B** The ankle joint line orientation (AJLO) measured as the tibial plafond to horizontal axis angle (TPHA; the red line represents the tangential line to the tibial plafond, and the green line is the horizontal axis)

**Fig. 2:**
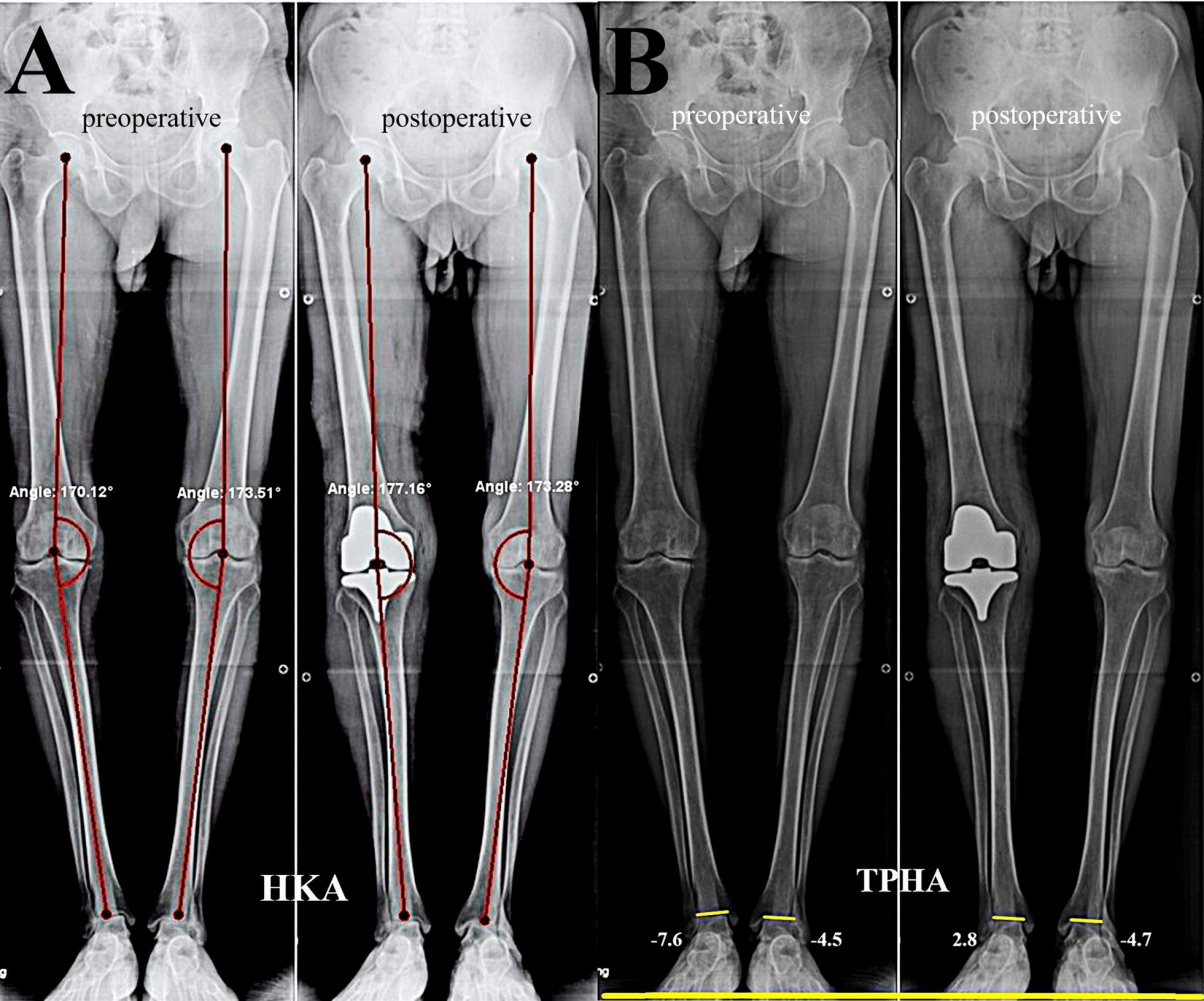
Male patient, 65 years old, who underwent right TKA. **A** The preoperative and postoperative hip-knee-ankle (HKA) angle evaluation. **B** The ankle joint line orientation (AJLO) measured as the tibial plafond to horizontal axis angle (TPHA) changes between pre- and postoperative stages

1. Radiological parameters (for both sides; Figs. 1 and 2)

  A To evaluate the lower limb mechanical alignment, we measured the hip-knee-ankle (HKA) angle as the medial angle between the femoral and tibial mechanical axes, where 180° was considered neutral, and varus and valgus deformities were considered when the HKA angle was less or more than 180°, respectively [31].
  B To evaluate the AJLO, the tibial plafond horizontal angle (TPHA) was measured as the angle between the tangent line to the tibial plafond and the ground level (representing the horizontal plane) [10, 13, 22, 32, 33]. The AJLO was considered neutral when TPHA was 0° (the tibial plafond line was parallel to the horizontal axis), while varus was given a negative value (indicated by a medial upward slope of the tibial plafond line), and valgus was given a positive value (indicated by a medial downward slope of the tibial plafond line) [22].
2. Clinical and functional parameters (for side A only):
  A The Knee Injury and Osteoarthritis Outcome Score (KOOS) was used to evaluate the knee joint [34, 35].
  B The American Orthopaedic Foot and Ankle Society (AOFAS) was used to assess the ankle joint [36].

The intraobserver reliability of the radiographic measurements was assessed using the intraclass correlation coefficients (ICCs). The same author remeasured 25 randomly selected radiographs 2 weeks after the initial evaluation. The ICCs demonstrated excellent agreement, with a value of 0.991 (95% CI 0.977–0.996).

### Statistical analysis

Statistical analysis was performed using the Statistics Kingdom online platform (https://www.statskingdom.com/index.html). The normality of data distribution was evaluated using the Shapiro–Wilk test. Data were presented as mean ± standard deviation (SD), median and interquartile range (IQR), or frequencies and percentages, as appropriate. Normally distributed data were compared using the independent or paired *t*-test, while the Wilcoxon signed-rank test was used for non-normally distributed data. The chi-squared (*χ*^2^) test was applied to compare categorical variables. Correlations between variables were assessed using Pearson’s correlation coefficient. A *p*-value of less than 0.05 was considered statistically significant. A simple linear regression analysis was performed to evaluate the relationship between changes in the HKA and AJLO angles. The regression model used the change in HKA (*X*) as the independent variable and the change in AJLO angle (*Y*) as the dependent variable. The slope and intercept were calculated, along with the 95% confidence intervals and the goodness-of-fit metrics.

## Results (Table 1)

**Table 1:** Radiological and functional outcomes of the included patients

Parameter		Side A (70 knees, 100%)	Side B (70 knees, 100%)	*P*-value
KL-OA grade*	**2**	3 (4.3%)	7 (10%)	0.296^§^
	**3**	27 (38.6%)	30 (42.9%)	
	**4**	40 (57.1%)	33 (47.1%)	
*Radiological outcomes (for both sides)*				
HKA	Preoperative^†^	167.49 ± 6.25 (152–179)	168.91 ± 6.91 (150–179)	0.203^¶^
	Last follow-up^†^	177.08 ± 4.39 (168.4–190)	NA	NA
	*P*-value	** < 0.001** ^¶^	NA	
AJLO	Preoperative^†^	−7.11 ± 5.44 (−18.30–7.22)	−6.65 ± 6.50 (−24.10–11.00)	0.329^¶^
	Last follow-up^†^	−1.10 ± 4.91 (−14.30–10.00)	−6.38 ± 6.10 (−19.00–9.40)	** < 0.001** ^¶^
	*P*-value	** < 0.001** ^¶^	0.970^¶^	
*Clinical outcomes (for side A only)*				
		Preoperative	Last follow-up	
*KOOS*^†^ (70 knees, 100%)		45.20 ± 14.94 (14.30–75.00)	75.72 ± 13.28 (42–91.70)	** < 0.001** ^¶^
*AOFAS* ^‡^ * (31 knees, 44.3%)*		70 IQR (65–75)	90 IQR (80–90)	** < 0.001** ^#^

Data presented as: ^*^numbers (percentage), ^†^ mean ± standard deviation (range), ^‡^ median (*IQR* interquartile range). Statistical tests used for comparison: *§*the chi-squared test, *¶* students *t*-test, *#*Wilcoxon signed-rank test. Bold numbers indicate statistical significance, which was set if the *p*-value was < 0.05

*KL* Kellgren–Lawrence, *OA* osteoarthritis, *HKA* hip-knee-ankle, *AJLO* ankle joint line orientation, *KOOS* Knee injury and Osteoarthritis Outcome Score, *AOFAS* American Orthopaedic Foot and Ankle Society

We included 70 patients (side A, 70 knees; side B, 70 knees) with a mean age of 61.76 ± 5.96 years (49–77 years); the majority were females (54, 77.1%), and the patients’ mean body mass index (BMI) was 29.15 ± 5.08 (20–37.89). The follow-up time had a median of 18 (IQR: 12.0–46.2) months and a mean of 26.26 ± 19.12 months. There was no difference regarding the OA degree according to the KL grading system or the preoperative HKA angle between both sides; the *p*-values were 0.296 and 0.203, respectively.

Radiological outcomes (for both sides): in side A, the HKA significantly improved at the last follow-up compared with the preoperative measurements (*p* < 0.001). Regarding the AJLO, no difference was found between both sides preoperatively (*p* = 0.329); however, at the last follow-up, in side A, the AJLO changed significantly into less varus alignment compared with the preoperative status (−1.10 ± 4.91° versus −7.11 ± 5.44°, respectively, *p* < 0.001). While in side B, the AJLO showed no significant changes (−6.38 ± 6.10° versus −6.65 ± 6.50°, respectively, *p* = 0.970).

Functional and clinical outcomes (for side A only): the KOOS and AOFAS showed significant improvement at the last follow-up compared with preoperative scores, 75.72 ± 13.28 versus 45.20 ± 14.94 (*p* < 0.001) and 90 (80–90) versus 70 (65–75; *p* < 0.001), respectively.

**Fig. 3:**
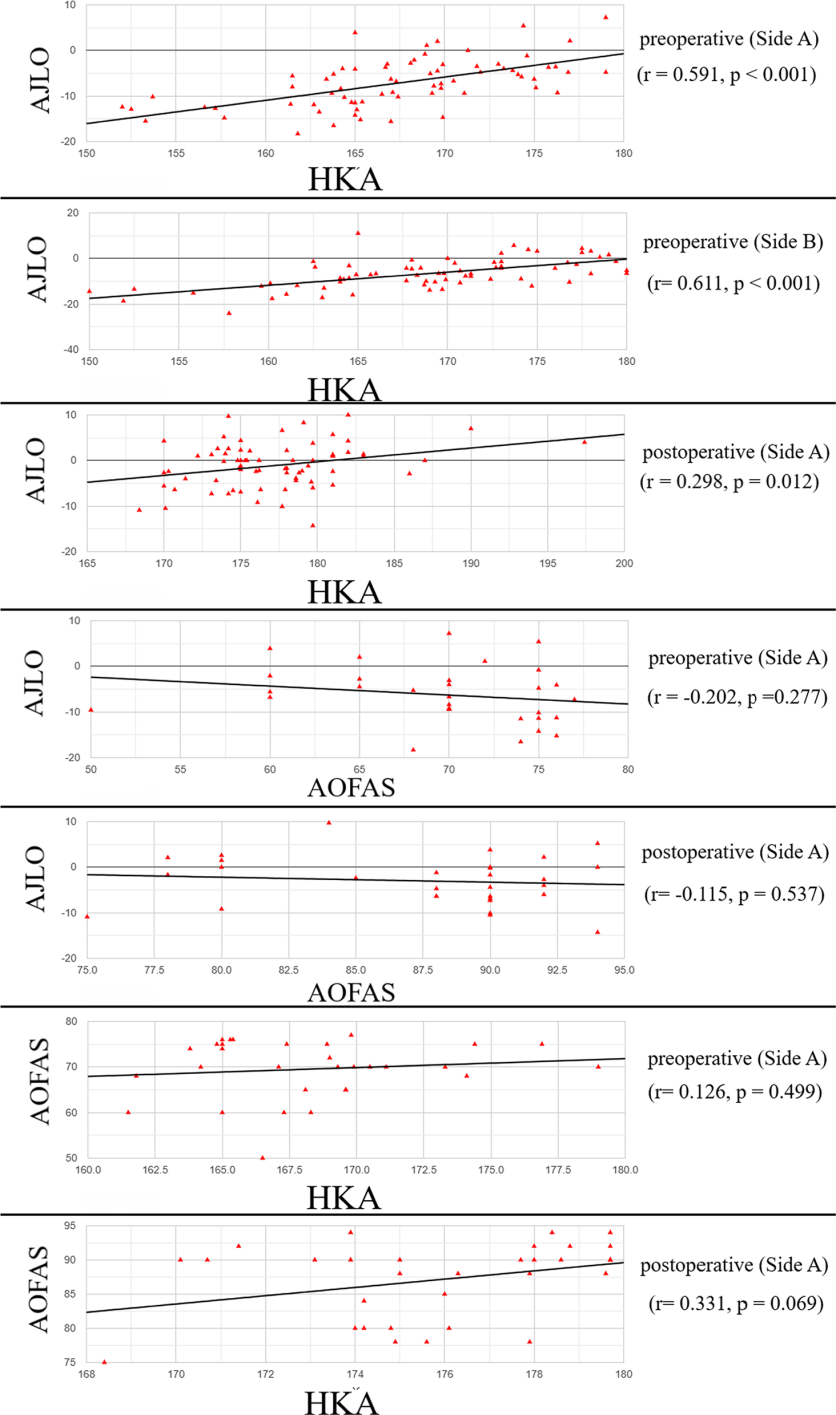
Correlation results between various variables, where side A is the operated side and side B is the non-operated side. *HKA* hip-knee-ankle angle, *AJLO* ankle joint line orientation, *KOOS* Knee Injury and Osteoarthritis Outcome Score, *AOFAS* American Orthopaedic Foot and Ankle Society

Regarding the correlations between various variables (Fig. 3), the preoperative HKA and AJLO on side A and side B showed significant positive correlations (*r* = 0.591, *p* < 0.001 and *r* = 0.611, *p* < 0.001, respectively). Furthermore, the postoperative HKA and AJLO on side A showed a significant positive correlation (*r* = 0.298, *p* = 0.012). The preoperative and postoperative AJLO and AOFAS on side A showed nonsignificant negative correlations (*r* = − 0.202, *p* = 0.277 and *r* = − 0.115, *p* = 0.537, respectively). The preoperative and postoperative HKA and AOFAS on side A showed nonsignificant positive correlations (*r* = 0.126, *p* = 0.499 and *r* = 0.331, *p* = 0.069, respectively).

**Fig. 4:**
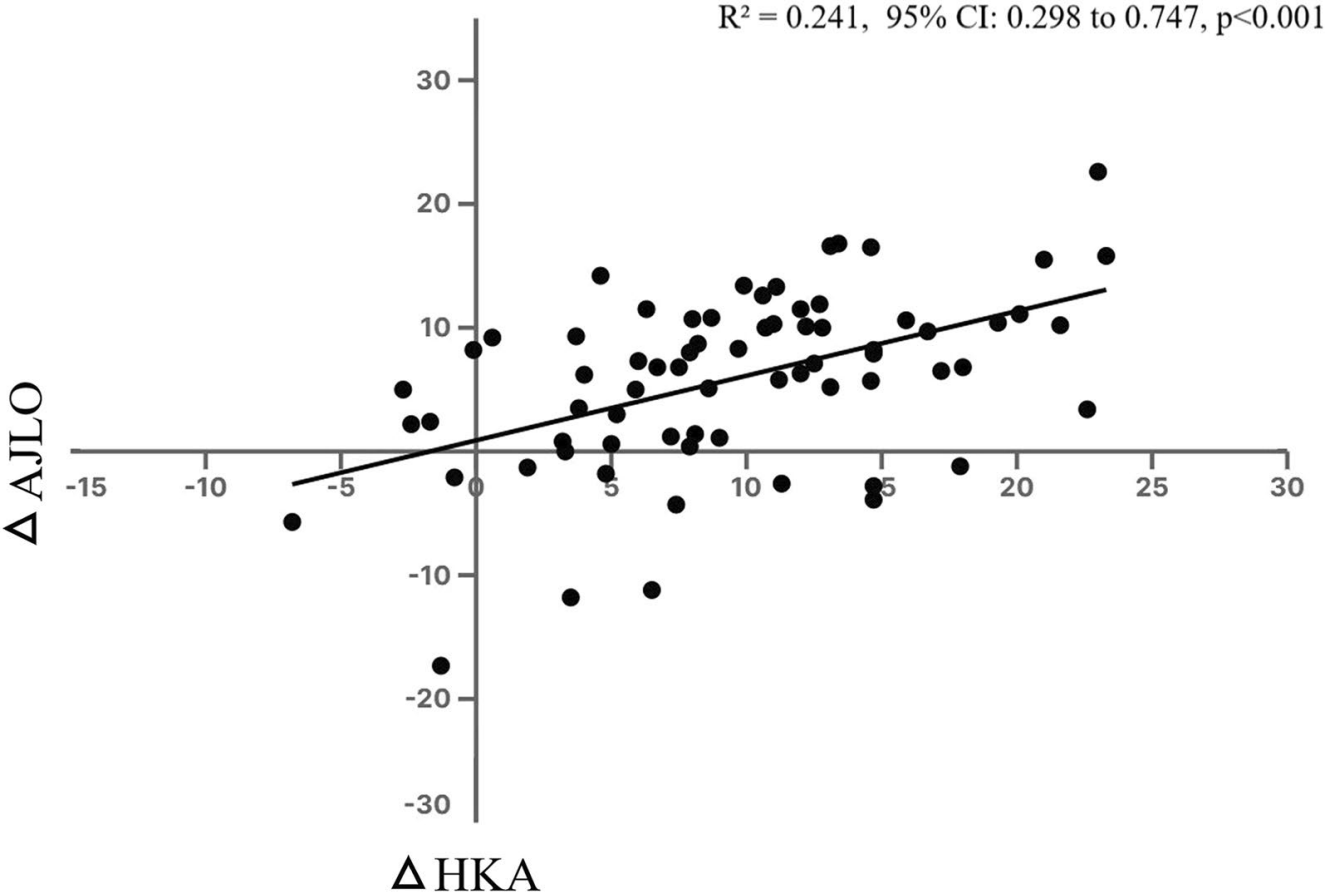
The linear regression result between ankle joint line orientation (AJLO) changes and hip-knee-ankle (HKA) angle changes

The linear regression analysis confirmed the statistically significant positive correlation between changes in AJLO angle (*Y*) and changes in HKA (*X*; *p* < 0.001), and the best-fit regression equation was *Y* = 0.5225*X* + 0.8943, indicating that for every 1° correction in HKA, the AJLO angle changed by approximately 0.5° (*R*^2^ = 0.241, 95% confidence interval: 0.298–0.747, *p* < 0.001; Fig. 4).

## Discussion

In an era leaning toward more personalized alignment options during TKA, surgeons should consider the whole lower limb as a single biomechanical unit, where changes at one joint (the knee) might affect the joints proximal and distal to it, the hip and ankle joints, respectively, and these changes should be anticipated and included in the preoperative planning protocol [11, 37, 38].

To the best of our knowledge, this is the first study to evaluate both ipsilateral and contralateral AJLO changes after ipsilateral TKA for knee OA with varus deformity. The current study’s most important findings are that correcting the knee varus deformity through TKA will lead to ankle joint realignment into less varus orientation on the ipsilateral side, while no compensatory changes will occur in the ankle on the contralateral side; furthermore, such AJLO post-TKA could be mathematically predicted. Although the ankle joint function improved post-TKA, this improvement was not significantly correlated to lower limb or ankle joint alignment changes.

Assessing the AJLO changes after TKA has been reported in previous studies, where most of the studies agreed that the ankle joint realigns after correcting the knee varus deformity; however, literature is diverse regarding how to assess the AJLO. In the current study, we used the TPHA, which was documented in previous studies to be a valid representative of the AJLO and correlated to changes in the lower limb coronal plane alignment [10, 13, 22, 32, 33].

Some previous reports assessed the AJLO as measuring the talar dome tangent line and horizontal reference lines [22, 39, 40]. In the current study, as we included patients with normal ankle joints, we hypothesized that the tibial plafond was parallel to the talar dome, as reported in literature [41]. This was confirmed in a study by Konrads et al., where the authors noted that the tibio talar tilt angle (TTTA) was 0° pre- and postoperatively in all of their 154 patients who underwent around-the-knee corrective osteotomies [13]. Alshrouf et al. evaluated the various angles to assess changes at the ankle level in response to lower limb corrective osteotomies; they measured the mechanical lateral distal tibia angle (mLDTA), mechanical malleolar angle (mMA), malleolar horizontal orientation angle (MHA), TPHA, and TTTA; the authors found that the TPHA changes correlate to lower limb alignment changes and concluded that TPHA is reliable to assess the AJLO in the coronal plane [32].

In a study by Lee et al. evaluating the changes occurring at the ankle joint after TKA, they included 142 knees (128 with varus deformity versus 14 with valgus deformity) in 111 patients having a mean age of 65.8 years. The authors reported a significant change in the TPHA from a preoperative mean of 10.0 ± 3.9° to a postoperative mean of 4.4 ± 4.0°, with a calculated overall mean difference of 5.6 ± 3.8° (they did not mention the direction of such deformity correction). Furthermore, the knees with varus deformities were corrected significantly from a mean preoperative varus of 8.5 ± 5.0° to a mean postoperative valgus of 4.7 ± 3.5°, with a calculated overall mean difference of 13.2 ± 5.1° in the varus angle [10]. In the current study, we achieved nearly similar correction degrees, where the AJLO was corrected by an overall mean of 5.9 ± 7.1°, and the knee varus deformity was corrected by an overall mean of 9.6 ± 6.7°.

We reported a significant positive correlation between HKA and AJLO both pre- and post-TKA; furthermore, the linear regression analysis showed that for each degree change in HKA, the AJLO would correct by 0.5°, coinciding with previous studies’ results. A study by Nazlıgü et al. evaluated the AJLO changes after correcting knee varus deformities by TKA at a mid-term follow-up (a mean of 32.50 ± 6.68 months) in 179 patients (204 knees) having a mean age of 65.57 ± 6.99 years. The knee alignment was evaluated using the HKA angle, while the TPHA was among the AJLO assessment angles. Comparing the last follow-up to the preoperative measurements, the authors reported significant improvement (*p* ≤ 0.05) in HKA (13.32 ± 4.83 versus 4.42 ± 2.97) and TPHA (9.61 ± 4.81 versus 4.46 ± 3.89), which indicates less varus AJLO at the last follow-up. They reported a statistically significant positive correlation between the increase in the HKA and the change in TPHA (*r* = 0.682); the authors reported that a 1° change in HKA would lead to a 0.483° change in the TPHA [23].

In a study by Graef et al. aiming at evaluating the degree of limb alignment correction after TKA at which patients might start having ankle joint symptoms, they included 99 patients with preoperative knee varus deformity. They reported a significant positive correlation between preoperative HKA and AJLO (*R* = 0.94, *p* < 0.05), indicating that a varus HKA is associated with varus AJLO, similar to the finding we obtained in the current study. However, contrary to our results, the authors reported no correlation between the postoperative HKA and AJLO (*r* = −1.3, *p* = 0.2) [15].

Hodel et al. [22] evaluated the relationship between the lower limb axial alignment and the AJLO in normal individuals; they included 60 lower limbs in patients having a mean age of 27.1 ± 10 years (20–67 years), they evaluated the AJLO as we described in the current study, where the mean AJLO did not differ on the basis of gender and was 0.5 ± 4.4 (−11.7–10.0), while the mean HKA angle was 1.7 ± 3.7 (−8.0–9.0). The authors reported a significant correlation between HKA and AJLO; furthermore, after a multivariate regression analysis, they reported that an increase in the HKA valgus values by 1° was associated with an increase in the AJLO valgus orientation by 0.5° (95% CI 0.2–0.7; *P* < 0.001).

It is worth noting that in the linear regression analysis results from the current study, the value of *R*-squared (*R*^2^) was 0.241, indicating that HKA could explain 24.1% of the variability of AJLO, and other factors might be involved in such variability. The same remark was reported by Hodel et al., where they reported *R*^2^ = 0.37; *P* < 0.001 after they considered the effect of femoral anteversion on AJLO besides the impact of HKA, where they found that a decrease in femoral anteversion by 1° was associated with an increase in the AJLO valgus orientation by 0.2° (95% CI 0.1–0.2, *P* < 0.001); they concluded that 37% of the changes in the AJLO could be explained by changes in the HKA and femoral anteversion [22].

Regarding the functional outcomes, Nazlıgü et al. reported significant improvement in AOFAS scores at the last follow-up (93.62 ± 7.54 versus 97.72 ± 5.21, *p* ≤ 0.05); however, they found a statistically significant negative correlation between the AOFAS scores in both HKA (*r* = −0.498) and TPHA (*r* = −0.233). We reported similar results in the current study where the postoperative AOFAS scores had a negative, yet nonsignificant, correlation with AJLO (*r* = −0.115, *p* = 0.537); however, on the contrary, it showed a positive correlation with HKA (*r* = 0.331, *p* = 0.069) [23].

At the last follow-up, Graef et al. reported a significant positive correlation between HKA correction degrees and the Foot Function Index (FFI; *R* = 0.91, *p* < 0.05), revealing that an increase in HKA aggravates the ankle symptoms. The authors further indicated that HKA correction of 14.5° is considered as a cutoff value for an increase in postoperative ankle pain (area under the curve (AUC) 0.88 (95% CI 0.808–0.958), sensitivity = 0.778, specificity = 0.889), and the odds ratio (OR) for ankle pain development was significantly increased beyond this cutoff value (OR = 15.6, 95% CI 3.2–77.2, *p* < 0.05) [15].

In a systematic review by Feng et al. [16], to understand the hindfoot and ankle joint behavior after correcting the varus knee deformity resulting from primary OA by performing TKA, 1157 knees from eight studies were included. Four studies reported on foot and ankle clinical outcomes; in two studies, the authors reported improved AOFAS scores after TKA (namely Okamoto et al. [6] and Palanisami et al. [42]), while in the other two, the authors reported that ankle pain increased after TKA (namely Kim et al. [9] and Chang et al. [4]), indicating the variability of knee deformity correction effect on the ankle functional and clinical outcomes.

As the lower limb acts as one mechanical unit, changes at any joint (hip, knee, ankle, and foot) could force the other joints to adapt with possible subsequent overload and early arthritic changes [43–47]. A typical example is the association between knee and ankle OA [10, 11, 19, 20]. In the current study, we included patients with normal ankle joints, so we could not judge the relationship between knee OA and ankle OA and whether correcting varus deformity has a role in modifying ankle complaints. However, we found it obligatory to allude to some previous studies on such issues.

The severity of ankle OA was associated with a moderate correlation with ankle joint malalignment, which is aggravated by knee coronal malalignment, according to Tallroth et al. [18]. Furthermore, the authors reported that about one third of the patients with knee OA included in their study had ankle degenerative changes. In a study by Huang et al. [8], the authors reported a relationship between varus knee deformity and various locations of ankle and foot pain, where the most common pain locations were the medial ankle (19.72%) and then the hindfoot (15.49%); in both locations, the pain incidence was higher as the varus deformity increased. The previously reported differences were not noticed in the midfoot and forefoot pain incidence. Lee et al. reported that 38.3% of the included knees with varus deformity had signs of ankle joint OA before TKA, and 21.8% developed ankle OA at the last follow-up after TKA (apart from the evident arthritic changes in the radiographs, the authors reported talar tilt differences), which might be a source of pain after TKA [10].

Apart from the inherent weakness of the retrospective nature of the study, we have further limitations. First, the AOFAS score was not reported for the whole cohort (in some patients, we could not collect the preoperative scores), which might influence the correlation results’ robustness. Second, the variability in the follow-up period might affect the functional outcomes results by introducing time-dependent bias, which was caused by two patients with a relatively longer follow-up (60 and 65 months) compared with the whole cohort; however, for both patients, KOOS outcomes were reported but not AOFAS, which did not significantly influence the primary objective of the study. Third, it might have been beneficial to collect the outcomes at various follow-up time points to evaluate the trend of AJLO changes over time, especially as some authors suggested that after 6 weeks, no further adaptation at the ankle joint will occur [48]. Third, we did not evaluate the joints distal to the ankle level (subtalar and foot), which might have a role in the adaptation and realignment process, as reported by Jeong et al. [49]. Last, we did not evaluate the rotational profile of the lower limb and its possible effect, which was alluded to in a study by Hodel et al. [22].

In conclusion, after TKA for managing knee OA with a varus deformity, the ipsilateral ankle joint realigned to a less varus position with an estimated half a degree of realignment for each degree of HKA varus correction; however, the ankle joint on the contralateral non-operated side possessed no changes. Although the ankle joint function improved significantly after TKA, this improvement was not correlated to the HKA or AJLO changes.

## Data Availability

All the data are included within the manuscript; however, the raw data could be provided upon a written request sent to the corresponding author.

## Abbreviations

TKA: Total knee arthroplasty
OA: Osteoarthritis
AJLO: Ankle joint line orientation
AP: Anteroposterior
KL: Kellgren and Lawrence
HKA: Hip-knee-ankle
TPHA: Tibial plafond horizontal angle
KOOS: Knee Injury and Osteoarthritis Outcome Score
AOFAS: American Orthopaedic Foot and Ankle Society
ICCs: Intraclass correlation coefficients
SD: Standard deviation
IQR: Interquartile range
TTTA: Tibio talar tilt angle
mLDTA: Mechanical lateral distal tibia angle
mMA: Mechanical malleolar angle
MHA: Malleolar horizontal orientation angle
FFI: Foot Function Index
OR: Odds ratio
